# Unforeseen clonal evolution of tumor cell population in recurrent and metastatic dermatofibrosarcoma protuberans

**DOI:** 10.1371/journal.pone.0185826

**Published:** 2017-10-04

**Authors:** Ensel Oh, Hae Min Jeong, Mi Jeong Kwon, Sang Yun Ha, Hyung Kyu Park, Ji-Young Song, Yu Jin Kim, Jong-Sun Choi, Eun Hee Lee, Jeeyun Lee, Yoon-La Choi, Young Kee Shin

**Affiliations:** 1 Laboratory of Cancer Genomics and Molecular Pathology, Samsung Medical Center, Seoul, Korea; 2 Department of Health Sciences and Technology, SAIHST, Sungkyunkwan University, Seoul, Korea; 3 Laboratory of Molecular Pathology and Cancer Genomics, College of Pharmacy, Seoul National University, Seoul, Korea; 4 College of Pharmacy, Kyungpook National University, Daegu, Korea; 5 Research Institute of Pharmaceutical Sciences, College of Pharmacy, Kyungpook National University, Daegu, Korea; 6 Department of Pathology and Translational Genomics, Samsung Medical Center, Sungkyunkwan University School of Medicine, Seoul, Korea; 7 Molecular Medicine and Biopharmaceutical Sciences, Graduate School of Convergence Science and Technology, Seoul National University, Seoul, Republic of Korea; 8 Department of Pathology, Changwon Samsung Hospital, Sungkyunkwan University School of Medicine, Changwon, Korea; 9 Division of Hematology-Oncology, Department of Medicine, Samsung Medical Center, Sungkyunkwan University School of Medicine, Seoul, Korea; German Cancer Research Center (DKFZ), GERMANY

## Abstract

Dermatofibrosarcoma protuberans (DFSP) is a very rare soft tissue sarcoma, generally of low-grade malignancy. DFSP is locally aggressive with a high recurrence rate, but metastasis occurs rarely. To investigate the mechanism of metastasis in DFSP, we analyzed the whole exome sequencing data of serial tumor samples obtained from a patient who had a 10-year history of recurrent and metastatic DFSP. Tracking various genomic alterations, namely somatic mutations, copy number variations, and chromosomal rearrangements, we observed a dramatic change in tumor cell population during the occurrence of metastasis in this DFSP case. The new subclone that emerged in metastatic DFSP harbored a completely different set of somatic mutations and new focal amplifications, which had not been observed in the primary clone before metastasis. The *COL1A1*-*PDGFB* fusion, characteristic of DFSP, was found in all of the serial samples. Moreover, the break position on the fusion gene was identical in all samples. Based on these observations, we suggest a clonal evolution model to explain the mechanism underlying metastasis in DFSP and identified several candidate target genes responsible for metastatic DFSP by utilizing The Cancer Genome Atlas database. This is the first study to observe clonal evolution in metastatic DFSP and provide insight for a possible therapeutic strategy for imatinib-resistant or metastatic DFSP.

## Introduction

It is increasingly accepted that tumor progression is driven by sequential selection of more aggressive subclones known as “cancer-clone evolution” [1]. This is a process similar to Darwinian natural selection wherein individuals with certain variants of a trait survive and reproduce at a higher rate than others with less advantageous variants do. In this way, the population adapts to changes in external environment and evolves. In the context of cancer therapy, the evolutionary process causes treatment failure and recurrence. Several studies of drug resistant tumors from colon and lung cancer patients revealed that the genomic profiles of pre-treatment tumors and post-treatment tumors were not always concordant [2–5]. The resistant clones in colon cancer patients, who initially benefited from EGFR blockade but experienced disease progression thereafter, often harbored mutations in *KRAS*, which were absent before the start of the therapy. Similarly, in cases of lung adenocarcinoma, the T790M mutation in *EGFR*, which was barely detectable in primary tumors, often became predominant after *EGFR* blockade. How tumor cells acquire the secondary mutations remains unclear. However, more sensitive detection methods increasingly suggest that these mutations exist in a small number of cells within the primary tumor population before treatment and then become the dominant clones owing to the drug selection pressure [4, 6]. These observations indicate that tumor evolution can affect therapeutic decisions and patient outcomes. Therefore, it is crucial to identify genetically distinct subclones at diagnosis.

In our clinic, we observed a dermatofibrosarcoma protuberans (DFSP) case that was initially benign, but then turned malignant and developed distant metastases during several recurrences over 10 years. DFSP is a rare cutaneous soft tissue sarcoma with distinct histologic features, including a storiform pattern of growth, spindle-shaped tumor cells, and CD34-positive staining [7]. DFSP is clearly identified by the characteristic and diagnostic chromosomal rearrangement of chromosomes 17 and 22, t(17;22)(q22;q13), which results in the fusion of the platelet-derived growth factor B (*PDGFB*) to collagen 1 alpha 1 (*COL1A1*) [8, 9]. The *COL1A1*-*PDGFB* fusion causes aberrant overexpression of *PDGFB*, a tyrosine kinase which acts as a potent growth factor and drives sustained activation of PDGF receptor signaling [10, 11]. The standard treatment of DFSP is a wide local excision that excises the tumor with a margin of tumor-free tissue around it. However, frequent local recurrences occur after a complete excision, although, surprisingly, metastases are extremely rare events with a probability of less than 5% [9, 12]. Based on the molecular mechanism of the *COL1A1-PDGFB* fusion, DFSP patients are often treated with a PDGFB receptor inhibitor, such as imatinib, but efficacy of this drug in a metastatic setting is still unclear because approximately 50% of metastatic DFSP cases show no response to imatinib [13–16]. A recent study of 263 DFSP cases investigated the expression patterns of metastatic DFSP and suggested EZH2 as a druggable target [17]. However, currently, there are few effective therapeutic options for imatinib-resistant or metastatic DFSP and the biological mechanisms of such cases remain poorly understood.

To investigate mechanisms underpinning metastatic DFSP, we collected serial tumor samples from a recurrent DFSP patient who experienced a transition from benign to malignant cancer over the course of several recurrences. The longitudinal collection of serial samples in combination with the next-generation sequencing (NGS) technology allowed us to track various molecular characteristics of metastatic DFSP at the genomic level. We observed a dynamic clonal evolution, which could be attributed to metastasis and drug resistance in this DFSP case. The present study is the first report to propose a genomic mechanism of metastatic DFSP and provides insight into the emergence of carcinogenic evolution in genetically distinct clones in primary DFSP tumors.

## Results

### Case history

**Fig 1:**
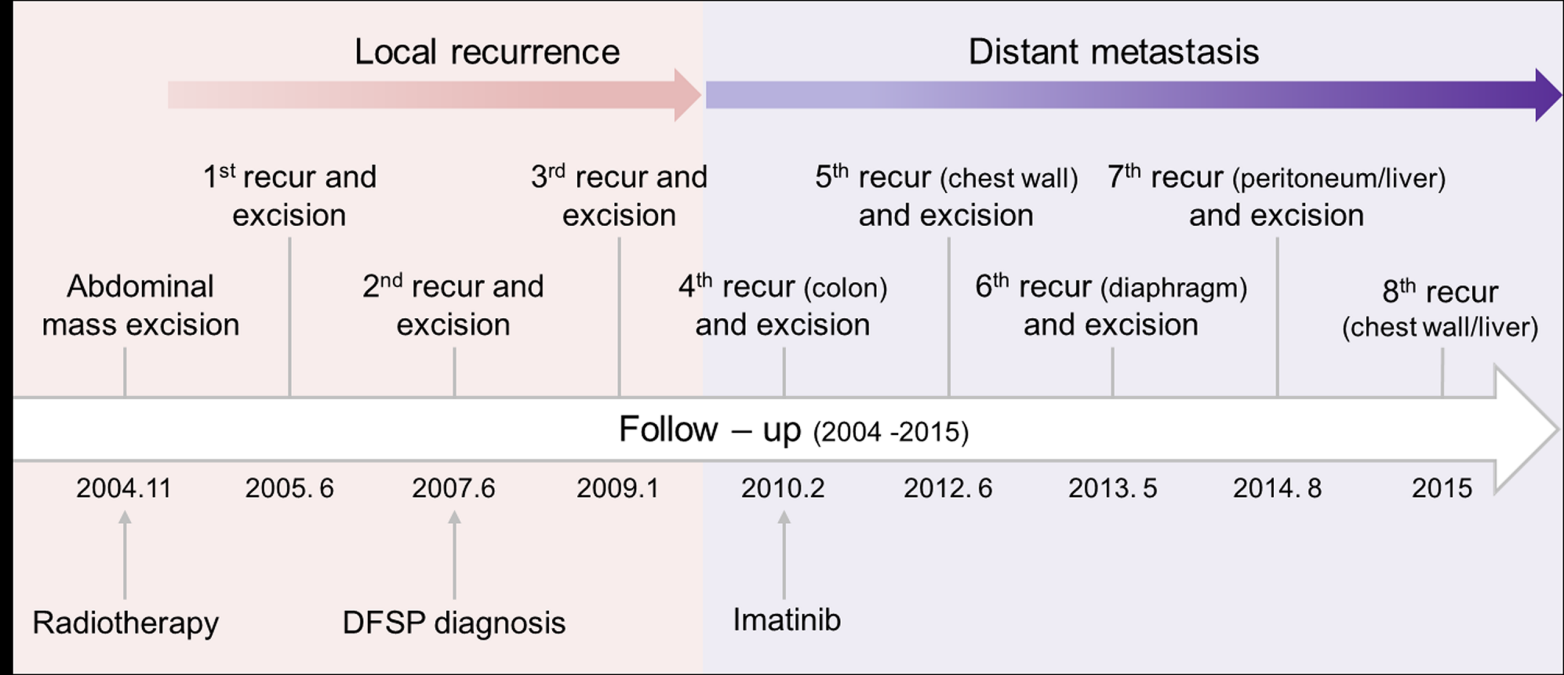
The case history of a patient with recurrent DFSP. The first tumor mass in the abdominal wall was resected in 2004 and recurrences have been occurring beginning in 2005 for over 10 years. Distant metastasis was first observed, and imatinib treatment was initiated, in 2010. Metastases occurred in other sites: chest wall (2012), diaphragm (2013), peritoneum/liver (2014), and chest wall/liver (2015).

A 52-year-old man with a suspected low-grade sarcoma in his abdominal wall had a surgical excision followed by radiotherapy for six weeks in 2004. In 2005, 2007, and 2009, local recurrences occurred and surgical excisions were performed for each recurrence (Fig 1). The tumor tissue in 2005 was a round white-tan mass with characteristic DFSP phenotypic features, such as storiform and infiltrative growth pattern and positive immunohistochemical (IHC) staining for CD34 (S1 Fig). The patient was diagnosed with DFSP in 2007 on the basis of the presence of *COL1A1-PDGFRB* fusion revealed by fluorescence *in situ* hybridization (FISH). The phenotype of the tumor in 2009 was similar to the one in 2007: a white-tan and round-shaped mass with a clear separation between tumor and normal tissue (S1 Fig).

Distant metastasis to the colon was first observed in 2010 (Fig 1). Unlike the previous tumors, the tumor observed in 2010 showed an irregular shape with unclear boundaries between the tumor mass and normal tissue, while the *COL1A1-PDGFB* fusion was sustained (S1 Fig). The patient began to receive imatinib treatment after resection in 2010, but metastasis continued to spread to other parts of the body, namely the chest wall (2012), diaphragm (2013), peritoneum, and liver (2014 and 2015) despite an escalation of the imatinib dose from 400 mg/kg to 800 mg/kg.

### Emergence of new tumor cell populations

**Fig 2:**
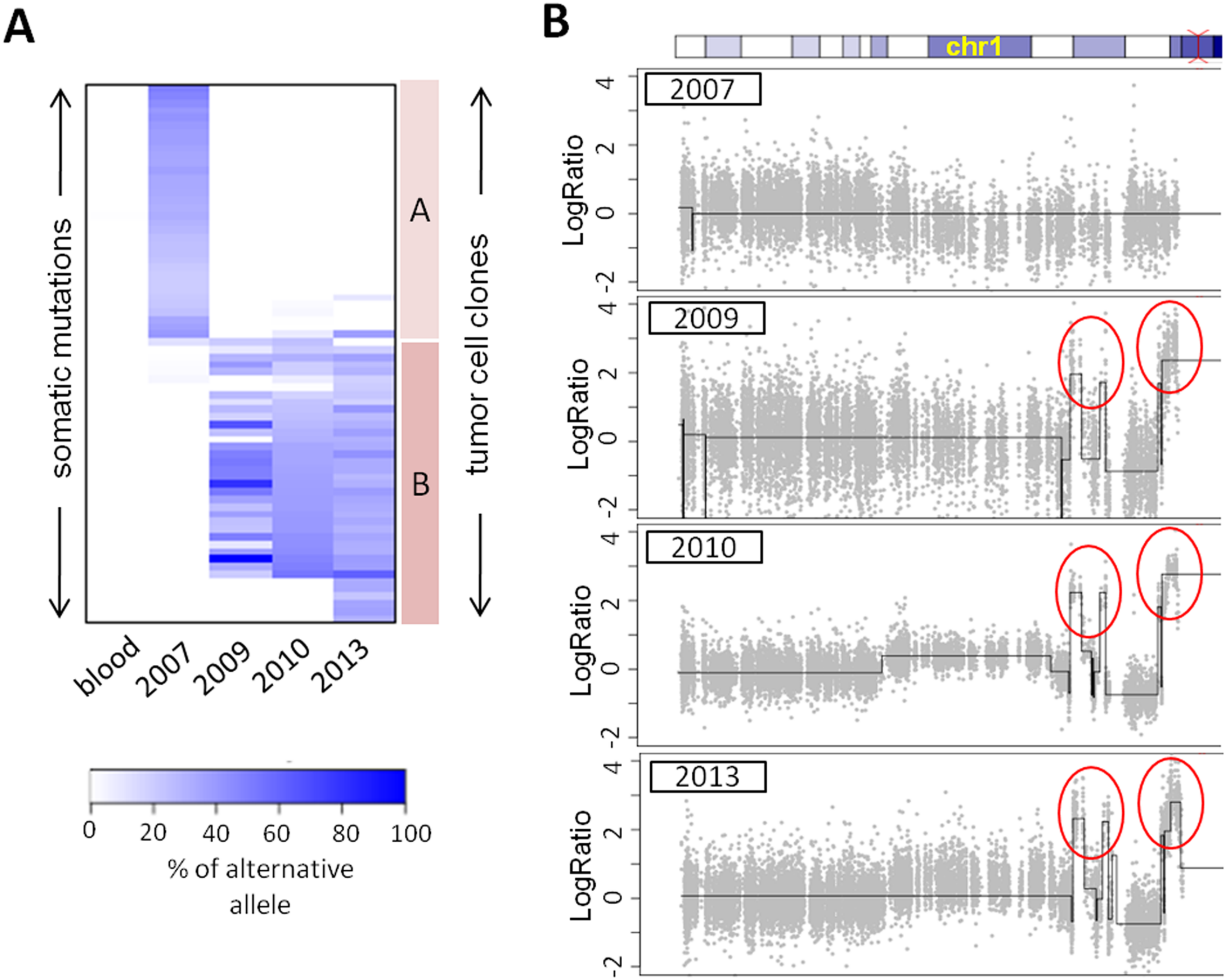
Somatic mutations and copy number variations. **A**. Heat map of somatic mutation calls. Alternative allele ratios are expressed by the color scale. Two types of tumor cell clones were identified by the somatic mutation profile. Only a small number of somatic mutations were shared by the two subclones. **B**. Copy number variation profiles of chromosome 1. Focal amplifications in chromosome 1 were found in the samples collected after 2007. DNA copy number is shown as the log2 value of the tumor/normal ratio.

We compared the somatic mutations and copy number variation profiles in the samples obtained at different time points in an attempt to find the genomic alterations that could be related to distant metastasis. To reduce false positive calls resulting from insufficient coverage, we selected competent variant calls supported by a minimum of 20 reads across all the samples (S1 Table). We identified two sets of mutations from the sequencing data of the serial samples, and they were almost always mutually exclusive (Fig 2A). In the sample from 2007, 37 somatic mutations/indels (revealed by the alternative allele ratio greater than 10%) were found. However, most of these mutations were rarely observed in the later samples. Conversely, nearly all the mutations observed in the later samples were not found in the sample from 2007. This indicates that there was a dramatic change in the tumor cell population between 2007 and 2009. In other words, clonal evolution occurred. The genetically distinct multiple clones could have arisen from intratumor heterogeneity rather than by clonal evolution. However, the likelihood of selecting the same subclones from three different samples would be extremely low. Sanger sequencing was then performed to detect 12 selected somatic mutations (eight in the sample collected in 2007 and four in later samples) in the corresponding samples from a different area that had not been used for whole exome sequencing (WES). The Sanger sequencing results showed mutually exclusive sets of mutations, the same as was found in the WES data (S2 Table). Copy number profiles also indicated emergence of a new tumor cell population. The samples from 2009 to 2013 harbored clear focal amplifications (1p22, 1p13, and 1p12) around the centromere area in chromosome 1. However, there were no such amplifications in the sample from 2007 (Fig 2B).

Taken together, our results suggest that there were genetically different tumor subclones during carcinogenesis in this DFSP case. The new subclone is likely associated with the development of metastasis because the emergence of the new tumor population and metastasis coincided. Moreover, the risk of metastasis remained as long as these metastasis-competent clones persisted. However, it was unclear when the new tumor cells arose, i.e., whether they were *de novo* populations or already present in the primary tumor. We found the answer from the analysis of chromosomal rearrangement described below.

### *COL1A-PDGFB* fusion

**Fig 3:**
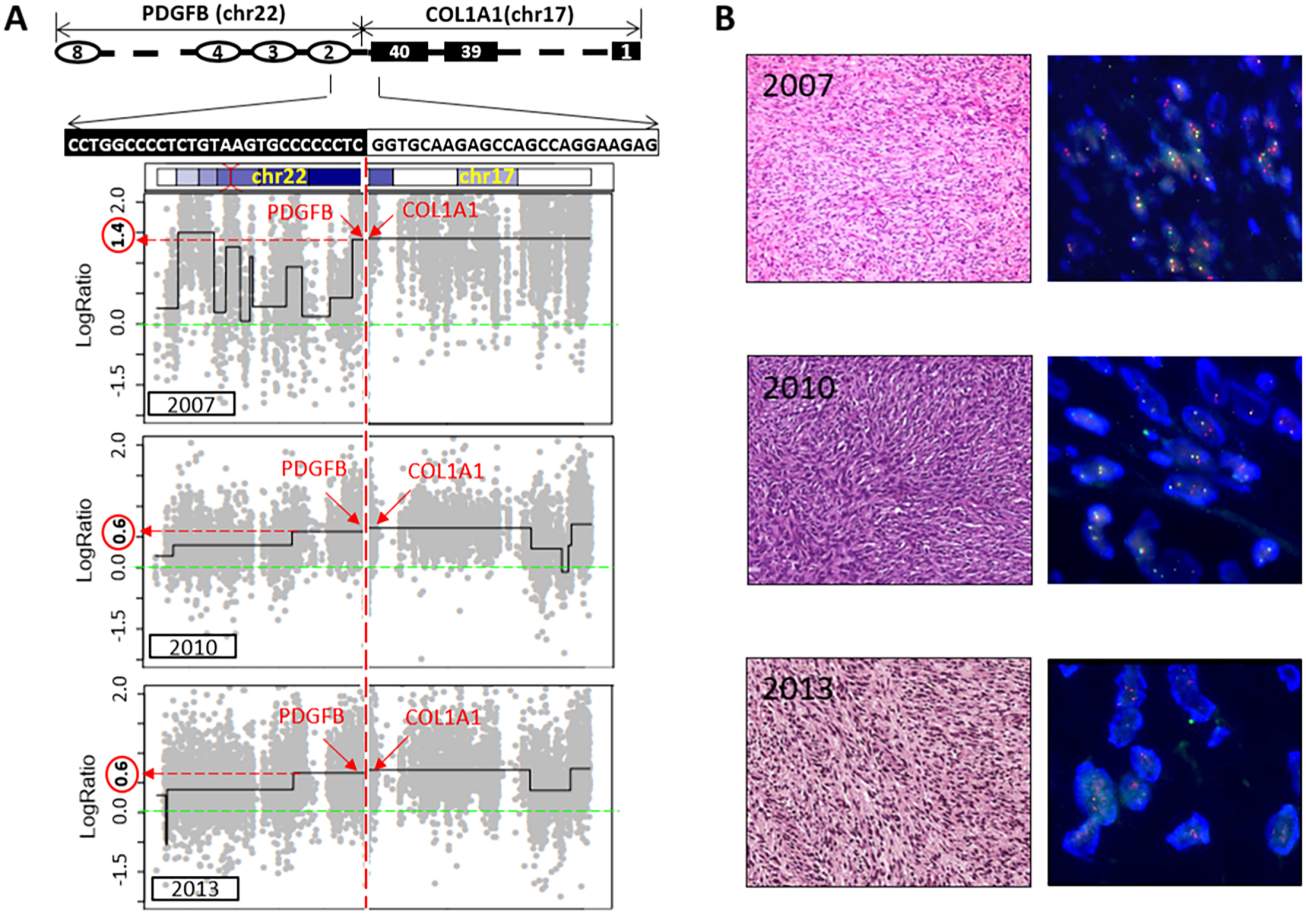
*COL1A1-PDGFB* fusion. **A**. The *COL1A1*-*PDGFB* fusion and copy numbers of the fusion gene. The breakpoint on the fusion gene was determined from WES data. All samples shared the same breakpoint, but copy numbers of the fusion gene were different in each subclone. The DNA copy number is shown as the log2 value of the tumor/normal ratio. **B**. Hematoxylin-eosin staining (left) and fluorescence *in situ* hybridization (FISH) using a *PDGFB* dual-color break-apart probe (right) of DFSP samples collected in 2007, 2010, and 2013. Histology of three tumor samples was overall similar and exhibited a characteristic spindle and whirring pattern. The 2010 tumor showed increased cellularity and the 2013 tumor displayed slightly increased cellular atypism. The mean copy number of the *PDGFB* gene (red dot) in the 2007 sample was 4.5 (range: 3–5), whereas in the 2010 and 2013 samples it was 2.8 (range: 2–3).

DFSP is characterized genetically by the translocation between chromosomes 17 and 22, which results in the *COL1A1*-*PDGFB* fusion. The distinctive fusion event was detected in all samples from this case. Surprisingly, the breakpoints of *COL1A1-PDGFB* were the same in all serial samples, regardless of subclone type, which harbored entirely different sets of mutations (Fig 3A). The presence of a common breakpoint clearly shows that the two genetically distinct subclones originated from a founder tumor cell in which a *COL1A1-PDGFB* fusion event occurred. It also indicates that the chromosomal rearrangement occurred before accumulation of the somatic mutations, so the chromosomal rearrangement is likely to be the earliest oncogenic event in the development of DFSP. Taken together, the common breakpoint shared by the two subclones demonstrates that the new tumor cells observed from 2009 were present in the primary tumor in 2007. In other words, there was a clonal evolution, where a subclone population existed at a low frequency in the primary tumor became the dominant clone, probably as a result of selective pressure.

Although the genetically distinct subclones shared a common DNA breakpoint, each subclone harbored different copy numbers of the fusion gene (Fig 3A). The copy number of the fusion gene in the sample from 2007 was estimated to be five copies (log2 ratio = 1.4), but this value was lower in the later samples (three copies; log2 ratio = 0.5). FISH results also indicated that the copy number of the fusion gene in the sample from 2007 was higher than in the later samples (Fig 3B). Amplification of *COL1A1-PDGFB* has been frequently observed in DFSP cases and the degree of amplification is thought to be related to DFSP subtypes and patient outcomes because the fusion gene is the major driving force in DFSP tumorigenesis. Fibrosarcomatous (FS)-DFSP is a subtype of DFSP, which is accompanied by the presence of the fusion gene, but carries an greater risk of recurrence and metastasis than does classic DFSP. It has been initially speculated that FS-DFSP may be characterized by a higher copy number of the fusion gene than classic DFSP. Later, it was shown that there was no statistically significant difference in the fusion gene copy numbers between FS-DFSP and classic DFSP, although these conclusions could be somewhat unreliable because of small sample sizes [18, 19]. However, the fusion gene copy number may affect the growth rate of each subclone in the beginning of DFSP tumorigenesis. The presence of the fusion gene provides a growth advantage, so the growth rate may accelerate if the fusion gene copy number increases. Consequently, the subclone that grows faster would eventually be dominant over other, more slowly proliferating subclones. In the present DFSP case, the subclone that harbored a higher copy number of the fusion genes was the major clone in the early primary sample before metastasis occurred.

### Tumor clonal evolution model

**Fig 4:**
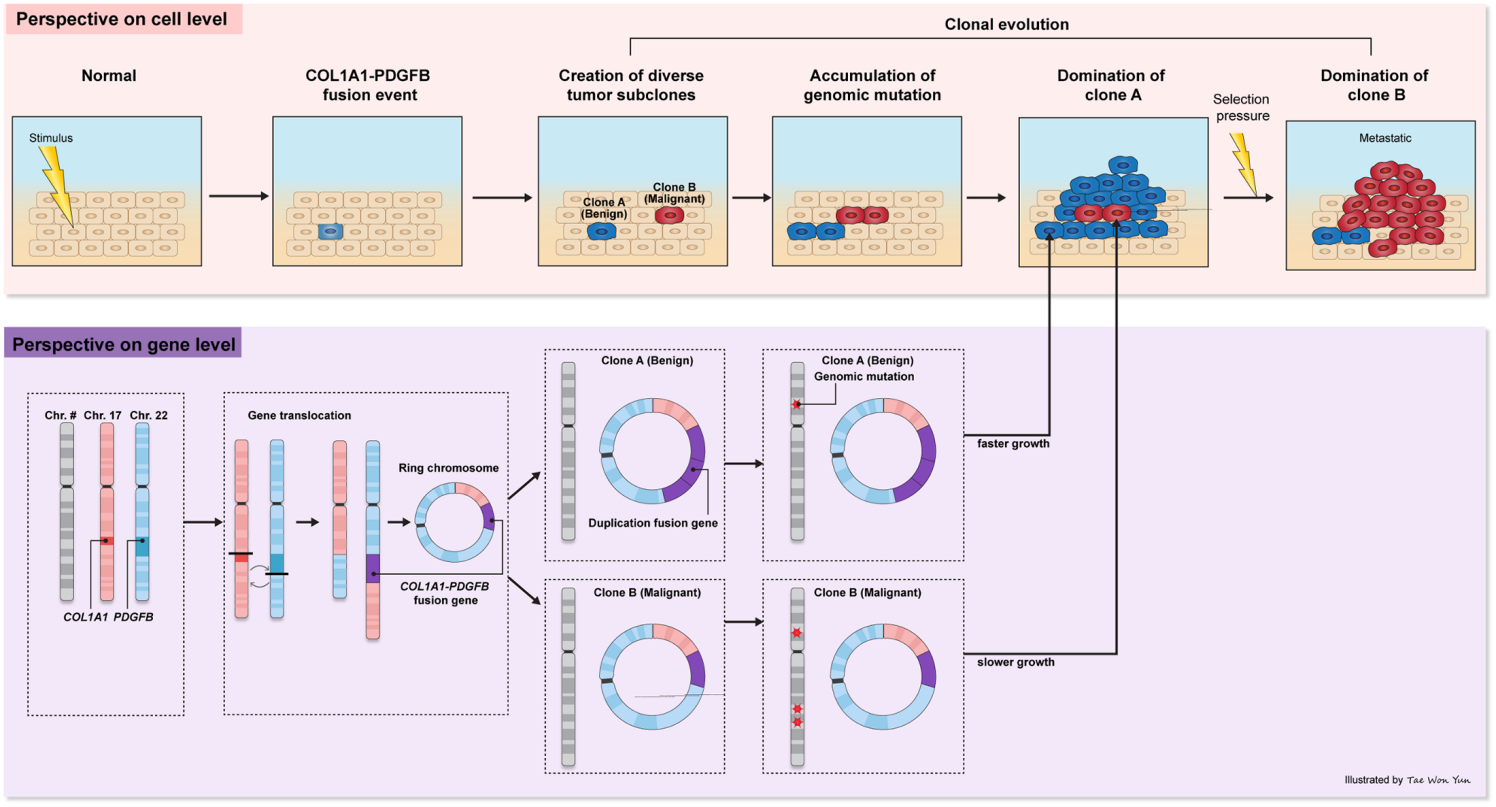
Clonal evolution model. *COL1A1*-*PDGFB* fusion initiates tumorigenesis of DFSP and diverse subclones arise through independent accumulation of somatic mutations. The primary subclone (Clone A), which is sensitive to the selection pressure, is gradually replaced by the expanding resistant secondary subclone (Clone B), which results in metastasis.

Based on the observation above, we built a clonal evolution model of the studied DFSP case (Fig 4). The common breakpoint in the *COL1A1*-*PDGFB* fusion gene clearly shows that the chromosomal rearrangement t(17;22) occurred at the very beginning of DFSP tumorigenesis and before accumulation of somatic mutations. Most commonly in DFSP, the chromosomal rearrangement breakpoint region is repeated in tandem within a supernumerary ring chromosome [20–22]. The ring chromosome bearing the *COL1A1*-*PDGFB* fusion gene would separate without centromere separation during mitosis and, subsequently, daughter cells are likely to have different numbers of the ring chromosome. The disparity in the copy number of the fusion gene would result in different growth rates in distinct subclones, and subclones with higher copy numbers of the *COL1A1-PDGFB* fusion gene would expand faster than other subclones, dominating the primary tumor mass. Moreover, abnormally rapid growth would be a driving force for genetic instability, which is thought to play a crucial role in enhancing genetic diversity in cancer. Somatic mutations occur independently in individual cells, generating multiple subclones with various traits that may be either advantageous or disadvantageous depending on physiological conditions. When exposed to a selective pressure, such as radiotherapy, chemotherapy, or specific drugs, the population of the major subclone decreases and the tumor mass becomes repopulated by other subclones that can survive under the selective pressure and succeed in spreading to other organs. According to our model, the acquisition of a driver mutation is a very early event of DFSP tumorigenesis and genomic instability created by the driver mutation results in the development of genetically distinct subclones.

### Therapeutic targets for metastatic DFSP

The clonal evolution coupled with metastasis suggests that the new subclone, which emerged in 2009, was responsible for metastasis in this DFSP case. To identify possible therapeutic targets in metastatic DFSP, we investigated the distinct genomic alterations found in the new subclone by utilizing The Cancer Genome Atlas (TCGA), a large public data repository which has sequencing data of more than 10,000 samples of various types of cancer.

First, we checked the frequency of the 21 mutations found in the new subclone in the TCGA database because frequent mutations are likely to play a crucial role in tumorigenesis. Second, we investigated the relative frequency of silent mutations within the 21 genes mutated in the new subclone. If a gene does not affect fitness, then there will be no selection pressure for it. Therefore, random mutations in such a gene are observed more often, i.e. the frequency of silent mutations is higher than in essential genes. In the TCGA database, well-known driver oncogenes show an extremely low frequency of silent mutations (~2–4%), whereas the average frequency of silent mutations is 27% on average per gene. We found that changes in the 21 mutated genes from our DFSP case were unique as none of these mutations was found in the entire TCGA database. However, all of the 21 genes mutated in the new subclone showed a high frequency of silent mutations, ranging from 20% to 30% in the TCGA database (Table 1). Taken together, the distinct mutations observed in the new subclone were thought to be passenger mutations that do not confer properties to an evolving cancer, which promotes metastasis.

**Table 1:** Frequency of 21 somatic mutations in TCGA data.

	Gene	Chr	Position	Reference allele	Alternative allele	AA_change	TCGA	
							Mutation freq	Silent mutation freq. (total mutation)
somatic mutations in metastatic DFSP (2009 ~)	*SPTA1*	chr1	158644387	T	A	E397V	0	24% (599)
	*ARID4A*	chr14	58831340	AA	-	E845fs	0	13% (98)
	*TRIM64C*	chr11	49075458	T	C	Y387C	0	0% (1)
	*SNTG1*	chr8	51351158	G	T	K73N	0	19% (103)
	*KBTBD8*	chr3	67054691	A	G	H434R	0	16% (69)
	*NF1*	chr17	29559773	G	A	S1124N	0	13% (352)
	*ACADVL*	chr17	7124251	G	T	D118Y	0	30% (33)
	*CSMD1*	chr8	4277581	G	A	S103L	0	25% (693)
	*TSNAXIP1*	chr16	67855068	C	T	R56C	0	9% (47)
	*FREM3*	chr4	144532457	A	T	V2001E	0	33% (33)
	*AGTPBP1*	chr9	88292383	T	C	I135V	0	22% (79)
	*PDILT*	chr16	20373877	T	G	M422L	0	31% (102)
	*SUCLG2*	chr3	67548643	T	G	D278A	0	33% (36)
	*SIDT1*	chr3	113329944	C	T	A604V	0	23% (76)
	*CCDC168*	chr13	103393536	C	A	M3170I	0	19% (124)
	*CREBBP*	chr16	3781373	C	T	R1626H	0	18% (267)
	*ZNF138*	chr7	64292048	A	T	K111N	0	36% (22)
	*GPR182*	chr12	57389165	C	T	A58V	0	21% (33)
	*TAS2R46*	chr12	11214052	A	-	W281fs	0	33% (15)
	*MBTD1*	chr17	49272648	T	C	Y433C	0	26% (31)
	*NGLY1*	chr3	25777563	G	T	D318E	0	17% (54)

Silent mutation rates of well-known driver oncogenes (total mutation)–*KRAS*: 2% (373), *BRAF*: 4% (536), *TP53*: 1% (2,273), *PIK3CA*: 2% (807), *EGFR*: 16% (303), *IDH1*: 2% (287), *NRAS*: 2% (186)

Next, we investigated the genes within the focal amplifications on chromosome 1, 1p22 (*OLFM3*), 1p13 (*CD58*, *MAN1A2*, *TBX15*, *WDR3*, and others), and 1p12 (*PHGDH*, *NBPF7*, *NOTCH2*, and others) because alteration of gene expression levels caused by DNA copy number variations can contribute to enhanced adaptive potential. TCGA data were used as a substitute for the sample obtained in 2007 (before metastasis), which was not available. The mRNA expression data of the sample from 2013 (after metastasis) was integrated into the TCGA expression database. Among all genes in the focal amplifications, only five (*TBX15*, *NOTCH2*, *PTGFRN*, *CNN3*, and *PHGDH*) showed appreciably higher expression levels than those observed in tumor samples from the TCGA database. In particular, the expression level of *TBX15*, which was negligible in most tumor types in the TCGA database, increased by approximately 100 times in this metastatic DFSP case (Table 2). The fold-change of *TBX15* is compatible to that of the driver oncogene *PDGFB*, whose expression level increased approximately 110-fold by both fusion and amplification.

**Table 2:** Expression levels of candidate genes’ mRNAs within the amplified regions.

		Amplification					Amplification + Fusion	
		*TBX15*	*NOTCH2*	*PTGFRN*	*CNN3*	*PHGDH*	*COL1A1*	*PDGFB*
	size (bp)	3,495	11,466	6,160	2,112	2,015	5,927	3,377
	fold change (DFSP/SARC) *	×98	×8	×8	×5	×2	×2	×110
	DFSP (2013)	274.24	167.00	219.51	646.67	151.41	2855.06	722.37
TCGA	SARC (n = 107)	2.80	21.22	28.72	133.00	67.87	1300.00	6.54
	BRCA (n = 1172)	1.74	25.29	22.71	101.10	24.07	837.10	12.52
	LUAD (n = 548)	1.22	15.78	13.77	84.52	9.16	283.80	6.42
	COAD (n = 477)	0.11	7.18	20.51	58.55	19.57	255.20	4.38
	SKCM (n = 375)	0.71	19.28	15.62	86.20	50.82	199.10	4.00
	HNSC (n = 541)	0.77	12.33	48.79	45.63	19.70	371.30	5.58

Gene expression levels were estimated in RPKM values.

Gene expression levels are median expression levels of the gene in the corresponding tumor type.

*Fold change of the gene expression level was obtained by dividing the median expression level of the gene in the DFSP case with the median gene expression level in the sarcoma data set (SARC) in TCGA.

**Fig 5:**
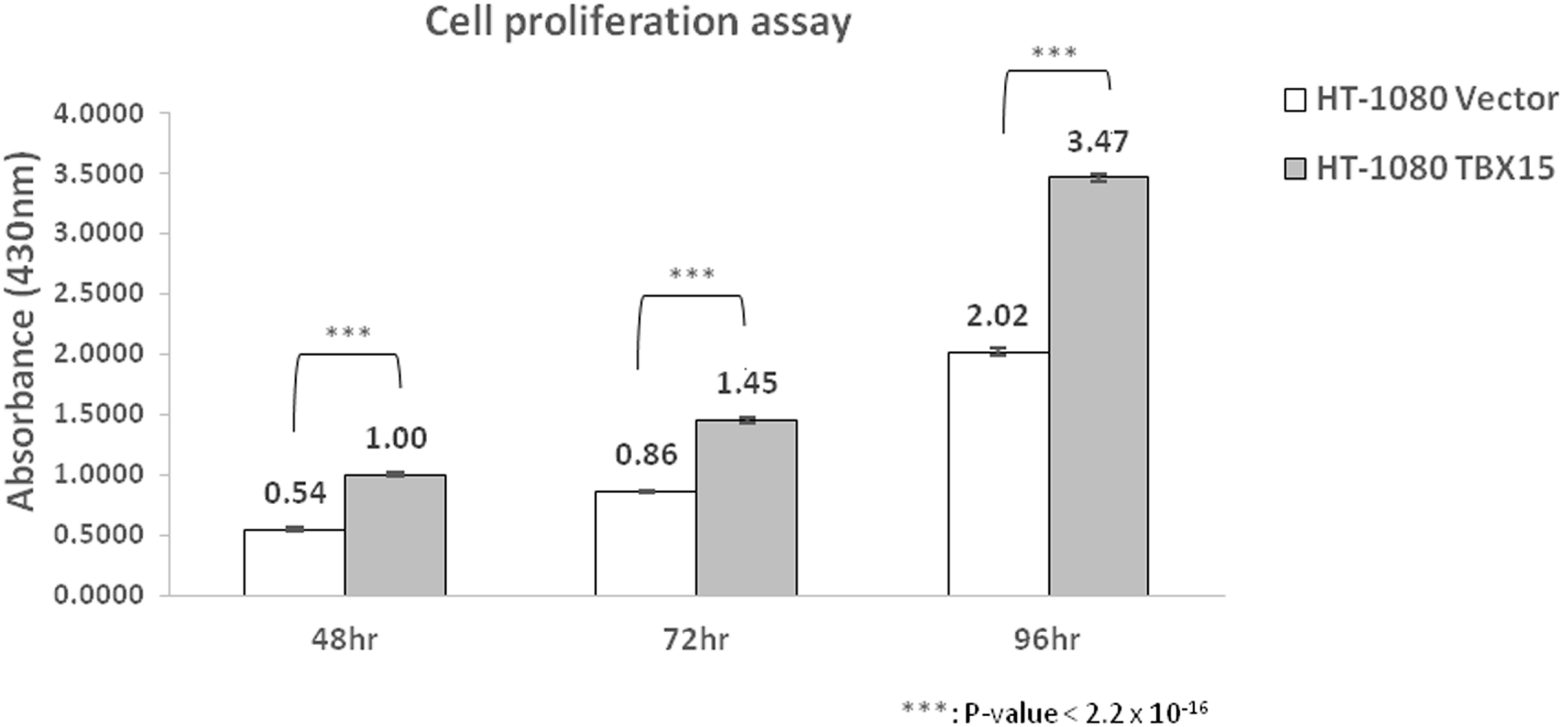
Cell proliferation assay of *TBX15* overexpression HT-1080. Cell proliferation of vector control and *TBX15* overexpressing HT-1080. Cells overexpressing *TBX15* grew faster than control cells.

To investigate oncogenic function of *TBX15* in DFSP, we performed an *in vitro* experiment using the fibrosarcoma cell line HT-1080. Three independent proliferation experiments were performed in duplicate and the expression level of *TBX15* was measured by qRT-PCR (S2 Fig). The sequences of primers used are shown in S3 Table. Cell proliferation was assayed at three time points (48, 72, and 96 hr) after *TBX15* transfection and the HT-1080 with *TBX15* overexpression grew significantly faster than control cells (Fig 5).

## Discussion

Tumor recurrence and metastasis are major challenges for the successful treatment of cancer. Clonal evolution theory has contributed to our understanding of the mechanisms of resistance to current cancer treatment and tumor recurrence. Several clonal evolution models have been suggested to explain or predict the response to targeted cancer therapy [5, 23, 24]. Because tumor evolution is driven by sequential selection of more aggressive subclones, timely detection of resistant clones is essential for a reliable prediction of treatment responses and choice of therapy. In our DFSP case, genomic changes preceded morphological and pathological changes. Genomic changes had occurred before 2009; however, there were no appreciable morphological changes in the sample in 2009 and distant metastasis was not observed until 2010. This finding shows that continuous tracking of genomic alterations in cancer cases is necessary for predicting patient outcomes.

An important question in understanding cancer evolution during treatment is whether the drug resistant cells are present prior to treatment or generated by treatment. There are few studies that have identified resistance-conferring mutations in pre-treatment samples because it is challenging to definitively ascertain which subclones were generated during treatment and it is entirely plausible that the resistant subclones were present but were not detected. In this study, the primary clone in 2007 and the new subclone in the later samples shared the common breakpoint between *COL1A1-PDGFB* fusions. This clearly shows that the two subclones originated from a founder tumor cell and they differentiated at the very early stage of DFSP carcinogenesis before the distinct mutations accumulated. This strongly supports the notion that the new subclone already existed in 2007, but the subclone was not detectable until it sufficiently expanded. Heterogeneous subclones generated after radiation therapy may have already existed since 2004.

Another question is what drove the outgrowth of the pre-existing resistant subclone between 2007 and 2009. Cancer therapy is often considered a very defined and stringent selection pressure during the evolution of cancer, but the DFSP patient received surgical resections only without any chemotherapeutic treatment in between. One possible explanation is that the new subclone was not removed in the primary site during the surgical excisions, while the other subclone was almost completely eliminated. The metastatic subclone might have spread slowly to other organs from the early stage of carcinogenesis, then it expanded fast as the population of the other subclone was reduced by excision. The selection pressures that caused the change of clonal population in this case remain unsolved.

In this study, we tracked various molecular characteristics of serial samples of metastatic DFSP and observed a dramatic change in tumor cell populations concomitant with metastasis. Furthermore, our study identified candidate genes that contributed to the recurrence and metastasis of DFSP. *TBX15* was suggested as the most plausible candidate gene in our study. *TBX15*, a member of the T-box gene family encoding transcription factors, is known to play a significant role in development, as mutations in this gene cause developmental dysmorphic syndrome in human [25, 26]. Recent studies found that TBX15 has an antiapoptotic role and its expression is altered in cancer cells, indicating a role for TBX15 in cell proliferation and carcinogenesis [27, 28]. In this study, the overexpression of *TBX15* in the fibrosarcoma cell line HT-1080 increased cell proliferation, suggesting oncogenic function of *TBX15* in DFSP. To date, the function of TBX15 remain poorly understood and further investigations are required to understand the role of TBX15 in cell proliferation and survival related to carcinogenesis.

## Materials and methods

### Patient samples, IHC, and FISH

This study was approved by the institutional review board (IRB) of the Samsung Medical Center (Seoul, Korea) and performed in accordance with the principles of the Declaration of Helsinki. Written informed consent was obtained from the patient. Frozen or formalin-fixed, paraffin-embedded (FFPE) tissues and blood samples were obtained from the patient with clinical information.

IHC analysis was performed on FFPE sections using a monoclonal antibody against CD34 (E1284; Spring Bioscience, Fremont, CA). *PDGFB* FISH was performed to detect *PDGFB* rearrangements using a dual-color break-apart probe (Macrogen, Seoul, Korea) as described previously [29].

### Whole-exome sequencing data analysis

WES data on three samples collected in 2007, 2009, and 2010 correspond to FFPE-1, FFPE-2, and frozen of “pair4”, respectively, in our previous study [30]. WES was additionally performed on a tumor tissue sample collected in 2013 as well as on blood samples collected at the same time. The samples from 2010 and 2013 were fresh frozen samples and the samples from 2007 and 2009 were FFPE tissues (S1 Fig). Exome capture was performed using a NimbleGen exome 2.1M array, which targets the content of the Consensus CDS (CCDS) project, i.e. nearly 30,000 coding genes (36.5M) in the CRCh37/hg19 genome assembly in total. The captured DNA was sequenced on an Illumina HiSeq 2000 instrument, generating 2× 90 bp paired-end reads. Sequencing data generated along the Illumina pipeline were aligned against the UCSC hg19 assembly using BWA (http://bio-bwa.sourceforge.net/) [31]. The aligned sequencing files were deposited in the Sequence Read Archive (https://www.ncbi.nlm.nih.gov/sra) with the project accession number PRJNA396980.

Somatic mutations and indels were called from each tumor sample by comparing sequences with those from matched blood samples using Varscan (http://varscan.sourceforge.net/). We selected somatic mutations with coverage ≥ 20× and alternative allele frequency of ≥ 20% in tumor and 0% in blood. The variants were annotated by ANNOVAR (http://www.openbioinformatics.org/annovar/). Copy number variants were detected by EXCAVATOR, a package developed for interpreting whole-exome sequencing data [32].

### Whole-transcriptome sequencing data analysis

Whole-transcriptome sequencing (WTS) was performed on the sample collected in 2013. The captured mRNA was sequenced using an Illumina HiSeq 2000 instrument, generating 2× 90 bp paired-end reads. The raw sequence files were deposited in the Sequence Read Archive (https://www.ncbi.nlm.nih.gov/sra) with the project accession number PRJNA396980. The GSNAP (http://research-pub.gene.com/gmap/) software was used to align the sequencing reads to the UCSC hg19 assembly [33]. Readcount (http://genome.sph.umich.edu/wiki/Bam_read_count) was used to count unique reads (MAPQ ≥ 20) at a gene level and the gene expression levels were normalized by reads per kilobase per million (RPKM). Fusion events were detected by FusionMap (http://www.arrayserver.com/wiki/index.php?title=FusionMap) [34].

### TCGA somatic mutation data analysis

We built a database from the latest somatic mutation data (.maf files, Feb 11, 2015) provided by The Cancer Genome Atlas (TCGA; https://tcga-data.nci.nih.gov/tcga/). This database accounts for 24 different types of tissue and 6,857 cancer patients. To reduce false positive calls resulting from insufficient coverage, we selected competent variant calls supported by a minimum of 20 reads and alternative allele ratio with a minimum of 0.2. More than 50% of the initial variant calls were filtered and the remaining 1,035,348 somatic mutation calls were used for the analysis. In the analysis, we included only the variants that affected protein-coding regions, i.e., missense, nonsense, insertion, deletion, and silent mutations. Other types of variants, which occurred in splice sites, UTR, or RNA, were excluded.

### TCGA gene expression data analysis

Raw read counts by gene in TCGA were converted into the reads per kilobase of transcript per million (RPKM) values to compare them with the expression data from the DFSP case. The fold change of gene expression was calculated by dividing median expression level in the DFSP case by the median expression level in sarcoma (SARC) data set in TCGA. As a reference, the expression levels of candidate genes in other types of cancers in TCGA, breast (BRCA), lung (LUAD), and colon (COAD), skin (SKCM), and head and neck (HNSC), are provided.

### Cell culture, DNA transfection and cell proliferation assay

HT-1080 cells were maintained with RPMI-1640 medium containing 10% FBS (Hyclone, Logan, UT, USA), 1% penicillin-streptomycin (Hyclone, Logan, UT, USA) under standard cell culture conditions. HT-1080 cells were transfected with pEF1α-AcGFP1-N1-TBX15 using Fugene HD transfection reagent (Promega, Fitchburg, WI, USA) in complete medium containing 5 ng/mL PDGF-BB (TONBO biosciences, San Diego, CA, USA). Cell proliferation of HT-1080 was measured by the WST assay using the EZ-Cytox Cell viability assay kit (Daeil Lab Service, Seoul, Korea).

## Supporting information

S1 Fig Case history of metastatic DFSP. **A**. Clinical information of the DFSP tissue samples. **B**. Surgically removed DFSP tumor masses. **C**. Histology of resected masses in the 2005, 2009, 2013, and 2014 samples.
(TIF)

S2 Fig *TBX15* overexpression in HT-1080. Ectopic expression of TBX15 in HT-1080 was verified by qRT-PCR. *HPRT* was used as reference gene.
(TIF)

S1 Table Somatic mutation calls. The list of somatic mutations which were selected by competent variant calls supported by a minimum of 20 reads across all the samples.
(XLSX)

S2 Table Sanger sequencing results of selected somatic mutations. Sanger sequencing results of 12 selected somatic mutations in corresponding samples from different area that had not been used for whole exome sequencing.
(XLSX)

S3 Table Primers information. The information of primers used in qRT-PCR.
(XLSX)
