# Supplementary figures and images for: Unforeseen clonal evolution of tumor cell population in recurrent and metastatic dermatofibrosarcoma protuberans
Source: PLoS One. 2017 Oct 4;12(10):e0185826. doi: 10.1371/journal.pone.0185826 (PMC5627939; doi:10.1371/journal.pone.0185826)

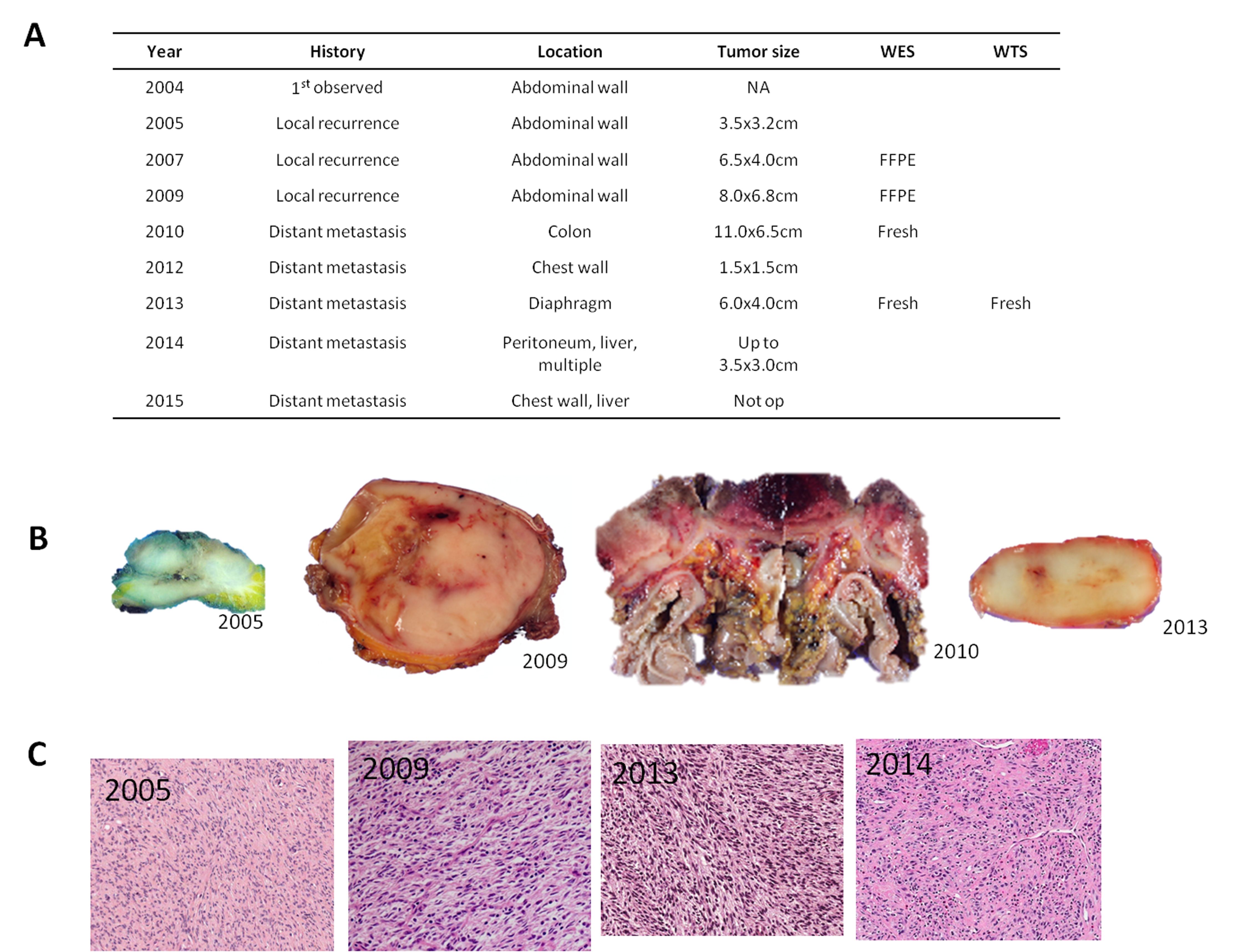

Supplement: S1 Fig — A. Clinical information of the DFSP tissue samples. B. Surgically removed DFSP tumor masses. C. Histology of resected masses in the 2005, 2009, 2013, and 2014 samples. (TIF) [file pone.0185826.s001.tif]

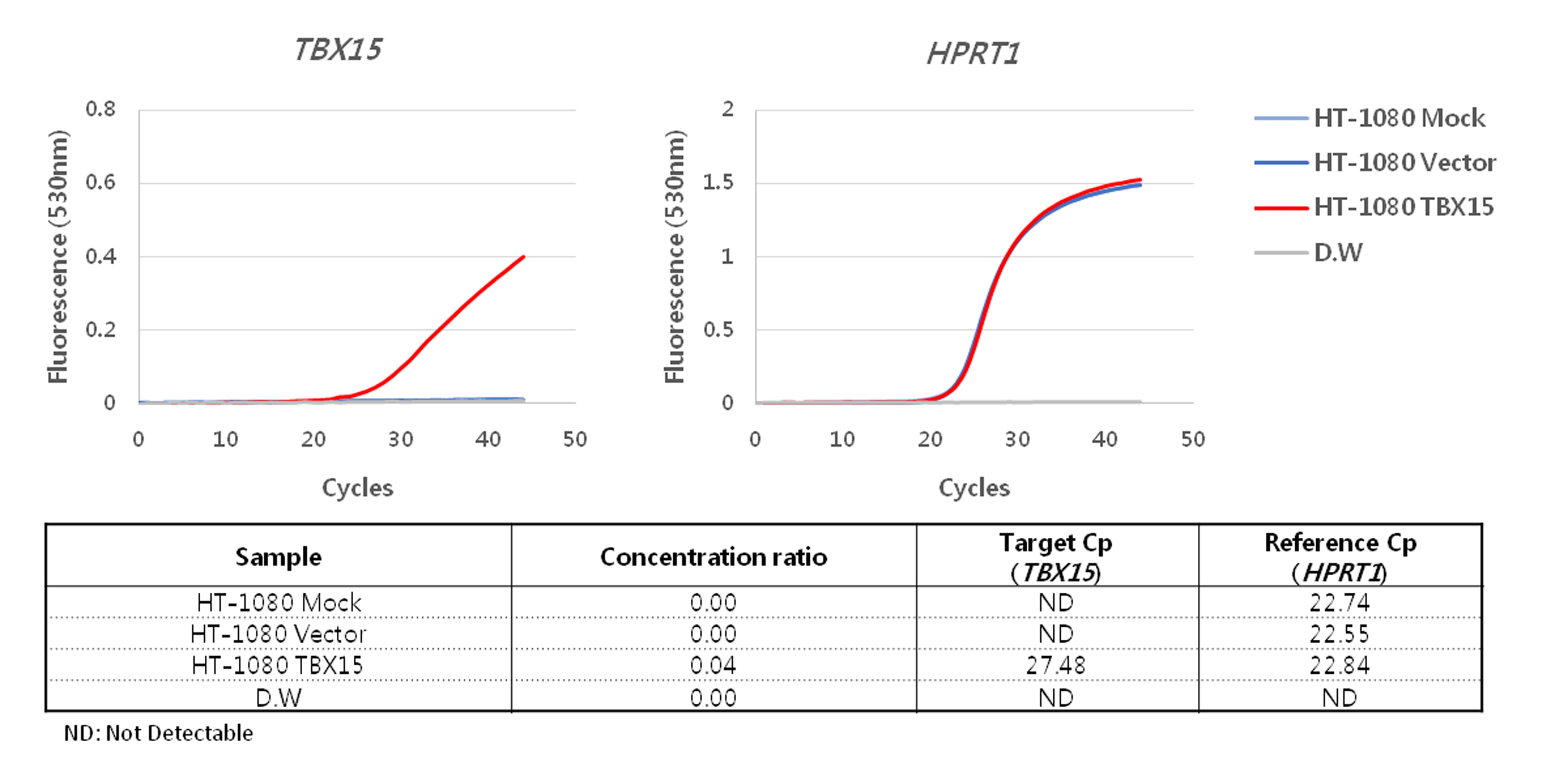

Supplement: S2 Fig — Ectopic expression of TBX15 in HT-1080 was verified by qRT-PCR. HPRT was used as reference gene. (TIF) [file pone.0185826.s002.tif]
